# *Shewanella oneidensis arcA* Mutation Impairs Aerobic Growth Mainly by Compromising Translation

**DOI:** 10.3390/life11090926

**Published:** 2021-09-06

**Authors:** Peilu Xie, Jiahao Wang, Huihui Liang, Haichun Gao

**Affiliations:** Institute of Microbiology, College of Life Sciences, Zhejiang University, Hangzhou 310058, China; 21907023@zju.edu.cn (P.X.); 22007025@zju.edu.cn (J.W.); 21607028@zju.edu.cn (H.L.)

**Keywords:** ArcA, regulation, aerobic growth, translation efficacy, peptide transportation

## Abstract

Arc (anoxic redox control), one of the most intensely investigated two-component regulatory systems in γ-proteobacteria, plays a major role in mediating the metabolic transition from aerobiosis to anaerobiosis. In *Shewanella oneidensis*, a research model for respiratory versatility, Arc is crucial for aerobic growth. However, how this occurs remains largely unknown. In this study, we demonstrated that the loss of the response regulator ArcA distorts the correlation between transcription and translation by inhibiting the ribosome biosynthesis. This effect largely underlies the growth defect because it concurs with the effect of chloramphenicol, which impairs translation. Reduced transcription of ArcA-dependent ribosomal protein S1 appears to have a significant impact on ribosome assembly. We further show that the lowered translation efficiency is not accountable for the envelope defect, another major defect resulting from the ArcA loss. Overall, our results suggest that although the *arcA* mutation impairs growth through multi-fold complex impacts in physiology, the reduced translation efficacy appears to be a major cause for the phenotype, demonstrating that Arc is a primary system that coordinates proteomic resources with metabolism in *S. oneidensis*.

## 1. Introduction

Two-component systems (TCSs) are a predominant form of bacterial signal transduction and transcriptional regulation in response to constantly changing external stimuli [1]. The Arc (anoxic redox control) system is an extensively studied TCS that primarily mediates transition from anaerobic to aerobic conditions and vice versa [2,3,4,5]. In *Escherichia coli*, the model organism for which our understanding of the physiological impacts and molecular mechanisms of Arc is most well developed, transmembrane sensor kinase ArcB undergoes autophosphorylation by sensing the redox state of quinone pool under anaerobic or microaerobic respiratory conditions, eventually resulting in the phosphorylation of DNA-binding response regulator ArcA (ArcA-P) through a phospho-relay mechanism [6]. Although ArcA-P functions primarily as a global repressor of aerobic metabolic pathways (the tricarboxylic acid (TCA) cycle in particular), Arc is now regarded as a global regulatory system, implicated in diverse biological processes with a regulon consisting of hundreds of target genes [7,8,9]. In bacteria equipped with Arc, Fnr (a fumarate–nitrate reduction regulator) and Crp (a cyclic–AMP receptor protein) are two other transcriptional regulatory systems profoundly involved in regulation of metabolism. Although Fnr is also a redox sensor, it differs from Arc in that it directly senses environmental oxygen with [4Fe-4S]^2+^ clusters [10,11]. The cAMP–Crp system is known to mediate carbon catabolite repression (CCR), a mechanism through which the synthesis of catabolic proteins for non-CCR sugar metabolism is inhibited when growing on CCR sugars such as glucose [12]. Since all of these three systems are deeply involved in metabolism, the terminal effects of their regulation are to alter growth rates. In recent years, a new mechanism has been proposed to illustrate growth phenomena mediated by cAMP–Crp [13]. It is suggested that the difference in the growth rates on CCR and non-CCR sugars arise from protein synthesis, which is the bottleneck of cell growth [12,13]. When CCR sugars are present, cells increase the expression of ribosomal and anabolic genes for rapid cell growth while reducing the expression catabolic genes for utilization of non-CCR sugars [13].

*Shewanella*, Gram-negative facultative anaerobes renowned for their remarkable respiratory abilities, have become a research model for investigating redox transformations of a variety of inorganic and organic chemicals, with *S. oneidensis* being the most intensively studied representative [14]. Given that respiration is the predominant means for energy production in *S. oneidensis* [15], global regulators mediating metabolic transition in response to the availability of different electron acceptors (EAs) have been investigated for decades and many surprising findings have been reported, particularly of Arc, Fnr and cAMP–Crp systems. Unlike *E. coli* Fnr, the *S. oneidensis* counterpart appears to have no significant role in metabolism regulation or even in general physiology [16]. On the contrary, it is evident that Crp (a cyclic-–MP receptor protein) is a commanding factor regulating respiration [17,18,19,20,21,22]. Although a Crp null mutant is severely impaired in the respiration of many non-oxygen EAs, it carries only a marginal defect in growth under aerobic conditions [17,19,22]. Interestingly, the influence of the ArcA loss on aerobic growth is much more significant in *S. oneidensis*, compared to the Crp depletion, implying that the Arc system is the predominant system mediating aerobic growth [23,24]. Multiple lines of evidence have shown that this aerobic growth defect of the ArcA null mutant (∆*arcA*) is partially due to compromised efficiency of oligopeptide metabolism [25,26].

Unlike the prototypical Arc system as observed in *E. coli*, the *S. oneidensis* counterpart is atypical because it uses two proteins (ArcS and HptA) to fulfil the role of the sensor kinase [23,25]. To date, the signal that is sensed by ArcS remains elusive. Despite this, it is clear that the *S. oneidensis* Arc system depends on the same phospho-relay mechanism for activation and recognizes highly conserved 15-bp DNA-binding motifs for controlling transcription of genes that follow [7,24,25,26]. Surprisingly, *E. coli* and *S. oneidensis* ArcA regulons differ from each other substantially; only 6 out of at least 50 members for the two ArcA regulons are shared, eliminating the possibility that the observed growth defect is due to interference with expression of the homologs of the established *E. coli* ArcA regulon members [23,24].

In addition to the slow aerobic growth, the *S. oneidensis* ∆*arcA* strain also carries a severe defect in the cell envelope integrity [27,28,29]. Both phenotypes are attributed to the ArcBA-mediated signal transduction and regulation because the manipulated production of ArcA but not ArcA^D54N^ (a mutant which could not be phosphorylated) corrects the two defects [24,27,28]. We have recently demonstrated that the cell envelope defect of the ArcA mutant is linked to activity of σ^E^ (RpoE), an alternative sigma factor which plays a primary role in mediating envelope biogenesis and envelope stress response [30], and appears independent of the aerobic growth defect [28,29]. The goal of this study was to unravel the mechanisms for the growth defect of the *S. oneidensis* ArcA mutant. We showed that the translation impairment resulting from the ArcA loss is likely the major cause for the growth defect. The ArcA mutation distorts the correlation between transcription and translation by down-regulating ribosome biosynthesis without interfering with ribosome assembly; such an effect is not observed with the lack of either Crp or Fnr. The reduced ribosome biosynthesis is partly due to the lowered expression of the *rpsA* gene, an ArcA regulon member that encodes the largest ribosome subunit. Thus, the *S. oneidensis* Arc system appears to play an important role in governing the allocation of resources toward protein synthesis and other aspects of cell growth.

## 2. Materials and Methods

### 2.1. Bacterial Strains, Plasmids, and Culture Conditions

A list of all bacterial strains and plasmids used in this study is given in Table 1. Information for primers used in this study is available upon request. Chemicals are from Sigma-Aldrich Co. unless otherwise noted. *E. coli* and *S. oneidensis* strains under aerobic conditions were grown in Lysogeny Broth (LB, Difco, Detroit, MI, USA) medium, which was modified to contain tryptone (10 g/L), yeast extract (5 g/L), and NaCl (5 g/L), at 37 °C and 30 °C for genetic manipulation. When needed, the growth medium was supplemented with chemicals at the following concentrations: 2, 6-diaminopimelic acid (DAP), 0.3 mM; ampicillin sodium, 50 µg/mL; kanamycin sulfate, 50 µg/mL; and gentamycin sulfate; 15 µg/mL.

### 2.2. Gene Knock In and Expression

The mutagenesis procedure for constructing in-frame deletion [34] was used to knock in the eGFP gene at the end of the rpll gene (the stop codon is removed during the mutagenesis). In brief, two fragments flanking the target sequences were amplified by PCR with outside primer containing *attB* and the gene specific sequence and inside primer containing the linker and the gene specific sequence. The PCR products of the first round were joined to both ends of the eGFP gene, generating the homologous fragment for knock-in with the eGFP gene centered. The fragments were introduced into plasmid pHGM01 by using Gateway BP clonase II enzyme mix (Invitrogen) according to the manufacturer’s instruction, resulting in mutagenesis vectors, which were maintained in *E. coli* DAP auxotroph WM3064. The vectors were subsequently transferred into relevant *S. oneidensis* strains via conjugation. Integration of the mutagenesis constructs into the chromosome was selected by resistance to gentamycin and confirmed by PCR. Verified transconjugants were grown in LB broth in the absence of NaCl and plated on LB containing 10% sucrose. Gentamycin-sensitive and sucrose-resistant colonies were screened by PCR for deletion of the target gene. Mutants carrying eGFP knock-in were verified by sequencing the mutated regions.

Growth of *S. oneidensis* strains studied in this study was measured by recording the optical density at 600 nm (OD_600_) values in triplicate with the wild-type as the control in MS defined medium with 30 mM L-sodium lactate [35]. When necessary, tryptone (BD Biosciences) was supplemented. For controlled expression in *S. oneidensis* strains, genes of interest generated by PCR were placed under the control of Isopropyl β-D-1-thiogalactoside (IPTG)-inducible promoter Ptac within pHGEN-Ptac [31]. After verification by sequencing, the resultant vectors in *E. coli* WM3064 were transferred into the relevant strains via conjugation.

### 2.3. Droplet Assays

Droplet assays were employed to evaluate growth inhibition on plates [36]. Cells of the mid-log phase were collected by centrifugation and adjusted to 10^9^ cfu/mL (colony forming unit), which was set as the undiluted (dilution factor 0). Ten-fold series dilutions were prepared with fresh medium indicated. A five microliter dose of each dilution was dropped onto LB or MS containing 0.5% tryptone plates containing required agents as indicated in the figure. The plates were incubated for 24 h or longer before being read. All experiments were conducted at least three times.

### 2.4. Microscopic Analysis

For visualization of GFP fusions, cells were processed with the protocol as described previously [33]. Slides were stored at 4 °C, and images were collected using a Zeiss ISM710 spectral two-photon confocal microscope (Carl Zeiss, Germany).

### 2.5. Analysis of Gene Expression

For qRT-PCR, *S. oneidensis* cells were grown in MS containing 0.5% tryptone with the required additives to the mid-log phase and collected by centrifugation, and RNA extraction was performed using the RNeasy minikit (Qiagen) as described before [37]. RNA was quantified by using a NanoVue spectrophotometer (GE Healthcare). The analysis was carried out with an ABI7300 96-well qRT-PCR system (Applied Biosystems). The expression of each gene was determined from three replicates in a single real-time qRT-PCR experiment. The Cycle threshold (*C_T_*) values for each gene of interest were averaged and normalized against the *C_T_* value of the *recA* gene, whose abundance is relatively constant during the log phase. The relative abundance (RA) of each gene was presented.

The activity of target promoters was assessed using a single-copy integrative *lacZ* reporter system as described previously [38]. Briefly, fragments containing the sequence upstream of the target operon were amplified, cloned into the reporter vector pHGEI01, and verified by sequencing. The resultant vector in *E. coli* WM3064 was then transferred by conjugation into relevant *S. oneidensis* strains, in which it integrated into the chromosome and the antibiotic marker was removed subsequently [18]. Cells grown to the mid-log phase under conditions specified in the text and/or figure legends were collected and β-galactosidase activity was determined by monitoring color development at 420 nm using a Synergy 2 Pro200 Multi-Detection Microplate Reader (Tecan), presented as Miller units.

### 2.6. Purification of Ribosomes

Ribosome extracts were prepared according to the established protocol [39]. *S. oneidensis* cells grown on MS supplemented with 0.5% tryptone to the mid-log phase were rapidly cooled and collected by brief centrifugation. The pellets were washed twice with and resuspended in 1× buffer A (10 mM Tris-HCl (pH 7.5), 60 mM KCl, 10 mM MgCl_2_) containing 0.5 mg/mL lysozyme and frozen overnight at −20 °C. The samples were thawed on ice the next day, treated with buffer A containing 0.5% Brij 58, 0.5% deoxycholate, and 0.1 unit/μL RQ1 DNase (Promega), and incubated on ice. Following incubation, ribosomal extracts were prepared by collecting the supernatant after brief centrifugation. Approximately 20 *A*_260_ units of the ribosomal extracts were layered onto a 10% to 40% linear sucrose gradient prepared in buffer B (10 mM Tris-HCl (pH 7.5), 50 mM NH_4_Cl, 10 mM MgCl_2_, 1 mM dithiothreitol (DTT)) by using an automatic Teledyne Isco gradient former and centrifuged at 25,000 rpm for 15 h at 4 °C in an SS34 rotor (Thermo Scientific). Following centrifugation, ribosomal fractions were collected from the bottom of the centrifuge tubes to the top by piercing the bottom of the tube and allowing samples to drip into collection tubes. *A*_256_ values were monitored using a Nanovue spectrophotometer (GE Health). The absorption of 1.0 at 256 nm corresponds to a concentration of 23 nM. Ribosome profiles were generated by plotting *A*_256_ values versus fraction numbers, using GraphPad Prism 8.4.

### 2.7. Electrophoretic Motility Shift Assay (EMSA)

Expression and purification of His-tagged *S. oneidensis* ArcA, and EMSA have been described before [24]. In brief, phosphorylation of purified ArcA was performed in buffer containing 100 mM Tris/HCl (pH 7.0), 10 mM MgCl_2_, 125 mM KCl, 50 mM dilithium carbamoyl phosphate for 60 min at room temperature. The probes used for EMSA were prepared by PCR with ^33^P end-labeled primers. The binding reaction was performed with ~25–50 fmol (~2–5 nM) labeled probes and various amounts of protein in 12 µL binding buffer containing 100 mM Tris/HCl (pH 7.4), 20 mM KCl, 10 mM MgCl_2_, 2 mM DTT, 0.2 μg/μL poly(dI·dC), and 10% glycerol at 15 °C for 60 min and resolved on pre-run 4.8% polyacrylamide native gels. Band shifts were visualized by autoradiography.

### 2.8. Other Analyses

Student’s *t* test was performed for pairwise comparisons. Values were presented as means ± standard error of the mean (SEM).

## 3. Results

### 3.1. The arcA Mutation Impairs Translation Efficacy

We have previously demonstrated that oligopeptide transport is partially accountable for the defect in aerobic growth of the *arcA* mutant, but its contribution is minor [26]. During the investigation, we noticed a significant difference in expression levels of the oligopeptide transport genes revealed by lacZ reporters and by qRT-PCR; the former are generally lower than the latter (Figure S1A,B). Given that the lacZ reporter assays the combined effect of both transcription and translation whereas qRT-PCR monitors transcription only, the difference suggests a possibility that the *arcA* mutation reduces translation efficiency. To test this notion, we set out to compare transcription and translation of a set of 12 genes that were chosen randomly in the wild-type and ∆*arcA* strains (Table S1). Among them, eight (*rplK*, *sspA*, SO_0783, *btuB*, *nuoA*, SO_2061, SO_3282, and SO4542) are predicted to be ArcA-independent with respect to transcription because of the lack of the ArcA-binding motif in their promoter regions. The remaining four genes are subjected to the direct control of ArcA. Promoters of all these genes were amplified and fused to the full-length *E. coli* lacZ gene with the same ribosomal binding site and the resulting constructs were integrated on the chromosome in the wild-type and ∆*arcA* strains.

**Figure 1:**
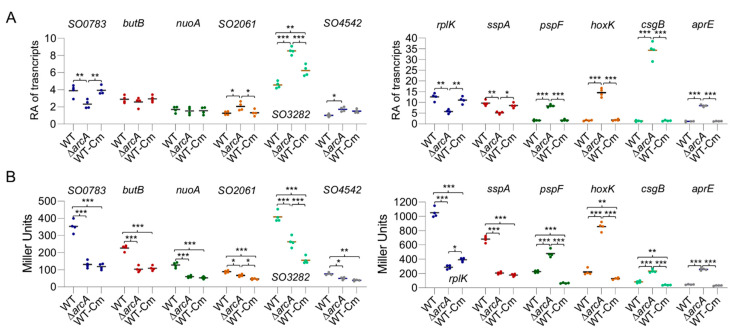
Impacts of the ArcA loss on transcription and translation. (**A**) Transcription levels of 12 genes revealed by qRT-PCR assay. Promoters of indicated genes were used to drive expression of the full-length *E. coli* lacZ gene integrated in the wild-type and ∆*arcA* strains, which were grown to the mid-log phase in MS containing 0.5% tryptone. The averaged values for each transcript were normalized to that of the *recA* gene, giving to relative abundance (RA) of transcripts. (**B**) Promoter activity assay. β-galactosidase activities in cells used in (**A**) were determined and presented as Miller units. Asterisks indicate statistically significant difference between values being compared (* *p* < 0.05; ** *p* < 0.01; *** *p* < 0.001).

We have previously shown that the wild-type and ∆*arcA* strains display a significant difference in growth in MS medium containing 0.5% tryptone under aerobic conditions (Figure S2) [40]. To be consistent, the same medium was used. Cells of the *S. oneidensis* strains under test grown into the mid-log phase were collected, from which mRNA levels of the lacZ gene were analyzed by qRT-PCR and protein levels were quantified by β-galactosidase activity. As shown in Figure 1A, mRNA levels of the lacZ gene driven by ArcA-independent promoters under test were either unaffected or slightly lowered in the ∆*arcA* strain. For promoters that are directly controlled by ArcA, we observed substantially altered abundances of lacZ transcripts between the wild-type and ∆*arcA* strains (Figure 1A). At protein levels, it was apparent that activities of LacZ driven by most of ArcA-independent promoters under test decreased significantly in the mutant strain although the overall trend was similar (Figure 1B).

**Figure 2:**
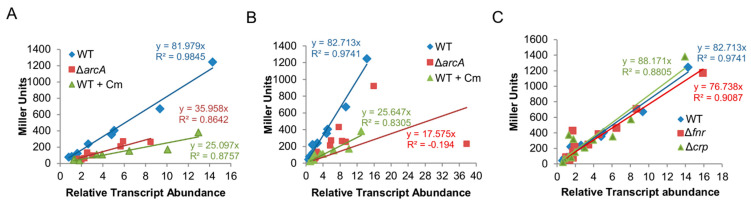
Correlations between transcription and translation. (**A**) Correlation between transcription and translation of 8 ArcA-independent genes in the wild-type and ∆*arcA* growth strains determined by linear regression from the data presented in Figure 1A,B. (**B**) Correlation between transcription and translation of all 12 genes (8 ArcA-independent and 4 ArcA-dependent genes) in the wild-type and ∆*arcA* growth strains determined by linear regression from the data presented in Figure 1A,B. (**C**) Correlation between transcription and translation of all 12 genes in the wild-type and ∆*arcA* growth strains determined by linear regression from the data presented in Figure S3A,B.

These data were then applied to statistics analysis to determine the relationship between the transcription and translation levels of the lacZ gene driven by promoters under test (Figure 2A). Values of the RNA/protein ratio derived from ArcA-independent promoters were fitted well to a linear regression model in both the wild-type and ∆*arcA* strains. Based on the slopes of regression lines, the *arcA* mutation induced a significant difference in the relationship between transcription and translation levels; there was reduction in the ratio of translation to transcription compared to that of the wild-type (Figure 2A). When data from ArcA-dependent promoters were included, the linear fitting for the ∆*arcA* strain was no longer statically confident (Figure 2B), In contrast, in the case of the wild-type, the impact of these data on fitting was negligible. These data manifest that the *arcA* mutation results in reduction in the ratio of transcription/translation in general but genes under the direct control of ArcA (at least some) are exceptional.

### 3.2. Reduced Ribosome Biosynthesis May Underlie Translation and Growth Defects of the S. oneidensis arcA Mutant

Translation is carried out via ribosome and there is a linear relation between the ribosome mass fraction and the growth rate [41]. This understanding resonates with our previous findings that the majority of components in protein synthetic machinery are concertedly down-regulated in both transcript and protein abundance [24,42] (Table S2). In addition, a remarkable number of translation-associated proteins, such as translation initiation and elongation factors and tRNA synthases, are present in less amount in the ∆*arcA* strain than the wild-type with respect to transcripts and proteins. On the contrary, most of members in transcription machinery are not affected by the ArcA loss. We therefore reasoned that compromised translation efficacy in the *arcA* mutant may be due to reduced ribosome contents.

**Figure 3:**
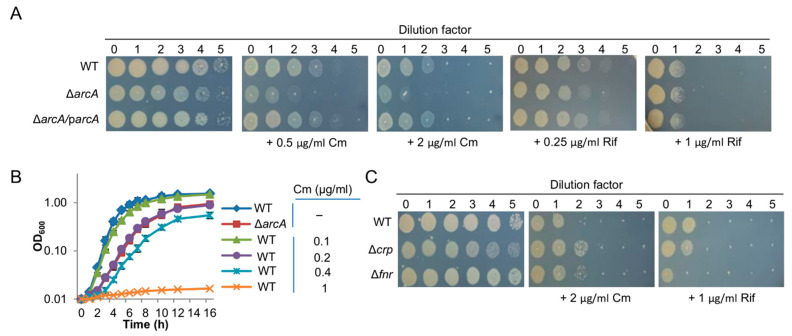
Impacts of antibiotics on *S. oneidensis* strains. (**A**) Susceptibility of the wild-type and *arcA* mutant strains to rifampicin (Rif) and chloramphenicol (Cm) determined by droplet assays on MS + tryptone plates. Cultures of indicated strains prepared to contain approximately 10^9^ cfu/mL were regarded as the undiluted (dilution factor, 0), which were subjected to 10-fold serial dilution. Five microliters of each dilution was dropped on indicated agar plates. Δ*arcA/*p*arcA,* the *arcA* mutant complemented by a copy of the *arcA* gene on a plasmid. (**B**) Impacts of chloramphenicol on growth of the wild-type strain. Growth of the wild-type and ∆*arcA* strains in MS containing tryptone without or with chloramphenicol at indicated concentrations. (**C**) Susceptibility to rifampicin (Rif) and chloramphenicol (Cm) determined by droplet assays on MS + tryptone plates. Experiments were performed independently at least three times, with representative results being presented or with the mean ± standard errors.

To test this, we compared susceptibility of the wild-type and ∆*arcA* strains for rifampicin and chloramphenicol, transcription- and translation-inhibition antibiotics respectively. We reasoned that the ∆*arcA* strain could be more susceptible to chloramphenicol because of the low abundance of ribosomes. The MICs of the wild-type for rifampicin and chloramphenicol were determined to be 0.25 and 1 µg/mL, respectively, which are in line with previous data [43]. As the growth defect of the ∆*arcA* strain interferes with MIC measurement, we used droplet assays for comparison. Clearly, rifampicin affected the wild-type and ∆*arcA* strains comparably (Figure 3A). On the contrary, the impacts of chloramphenicol were evidently more significant on the growth of the ∆*arcA* strain than that of the wild-type (Figure 3A).

We then attempted to mimic the effects of the *arcA* mutation on translation with chloramphenicol. A reduction in translation in a graded manner was achieved by using the antibiotic at sublethal doses; 0.2 µg/mL chloramphenicol decreased growth of the wild-type comparable to that of the ∆*arcA* strain in the absence of the antibiotic (Figure 3B). We then collected cells grown under this condition and assayed the transcription and translation of the set of 12 genes as described above. The transcription of most of them was not altered significantly compared to that derived from the untreated cells (Figure 1A). In contrast, the translation of all of these genes decreased in a concerted manner, including ArcA-dependent genes (Figure 1B). Moreover, data sets from both untreated and treated cells were fitted well to a linear regression model and, more importantly, the fittings of the *arcA* mutant and Cm-treated wild-type cells were similar (Figure 2A,B). These data support the theory that the *arcA* mutation compromises translation efficiency in *S. oneidensis*, likely by affecting ribosome biosynthesis.

### 3.3. ArcA (but Not Fnr or Crp) Impacts Translation Efficiency

A similar case of translation reduction resulting from the absence of a global regulator in *E. coli* has been reported: the cAMP-Crp regulatory system affects growth by coordinating proteome with metabolism in *E. coli* [13]. In *S. oneidensis*, Arc (rather than cAMP-Crp) is the primary regulator that is critically involved in the regulation of aerobic growth, and three global regulators Fnr, Crp and ArcA have been proposed to switch regulatory roles to some extent relative to their *E. coli* counterparts [19,20,21,24]. We therefore examined the effects of the Crp and Fnr loss on translation to determine whether the observation is specific to Arc in *S. oneidensis*.

Under aerobic conditions, the ∆*fnr* strain grew indistinguishably compared to the wild-type while the growth of the ∆*crp* strain was slightly impaired on MS supplemented with tryptone (Figure 3C). The same patterns were observed with both rifampicin and chloramphenicol (Figure 3C), indicating that the loss of either Fnr or Crp does not significantly alter the susceptibility of *S. oneidensis* cells to these two antibiotics. We then moved on to carry out the analysis of transcription and translation of the set of 12 genes in ∆*fnr* nor ∆*crp* cells. However, neither the ∆*fnr* nor ∆*crp* strain differed from the wild-type significantly in terms of transcription and translation of these 12 genes (Figure S3A,B). Importantly, linear regression models for RNA/protein ratio values derived from all these genes in these two mutants were highly similar to that of the wild-type but significantly different from that of the ∆*arcA* strain (Figure 2C). These data, all together, conclude that in contrast to the *arcA* mutation, the Fnr and Crp mutations do not distort the correlation between transcription and translation, eliminating the possibility that they affect ribosome biosynthesis to physiological relevant levels.

### 3.4. The ArcA Loss Reduces Ribosome Biosynthesis but Does Not Affect Ribosome Assembly

**Figure 4:**
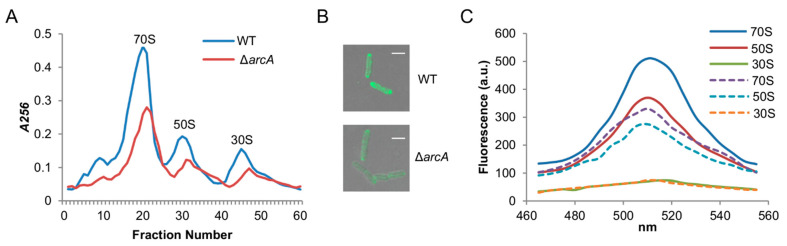
Impacts of the ArcA loss on ribosome biosynthesis and assembly. (**A**) Examination of ribosomal particles isolated by using the sucrose gradient centrifugation. Cells grown in MS + tryptone to the mid-phase was collected and subjected to ribosome isolation. Fractions collected from the bottom of the sucrose density gradient to the top. Ribosomal particles were identified by measuring the *A256* of rRNAs in each fraction. (**B**) Visualization of cells producing eGFP-tagged ribosomes with a confocal microscope. Scale bars, 1 µm. (**C**) Fluorescence spectra of 70S, 50S, and 30S ribosomal particles from the wild-type (solid lines) and ∆*arcA* (dash lines). Characteristic eGFP fluorescence was seen in 70S ribosomes and 50S subunits, but not in 30S subunits. Experiments were performed at least three times, with representative results being presented.

To validate that ribosome biosynthesis is impaired by the ArcA deletion, we assessed ribosome quantity in the wild-type and ∆*arcA* strains grown aerobically. From a similar number of cells grown to the mid-log phase, ribosomes were extracted and fractionated by sucrose gradient centrifugation to separate the 70S, 50S, and 30S complexes. Clearly, the ribosome profile of the ∆*arcA* strain was similar to that of the wild-type (Figure 4A). However, the averaged A260 value for ribosomal RNAs from the wild-type was 1.8-fold, based on the 70S complex, higher than that from the ∆*arcA* strain. These results support that the *arcA* deletion impaired ribosome biosynthesis without interfering with ribosome assembly.

For further confirmation, we used fluorescent protein-tagged ribosomes with eGFP-L9 fusion, whose *E. coli* counterpart functions fully [44]. The fluorescent recombinant L9 was constructed by fusing the eGFP gene with the *rplI* gene at its chromosomal loci. As expected, the L9 fusions in the background of the wild-type and *arcA* deletion had no deleterious effect on the growth rate relative to their respective parent strains (Figure S4). With a confocal microscope, we examined cells expressing eGFP-L9 fusions (Figure 4B). Clearly, the wild-type cells had a significantly stronger fluorescence than those of the ∆*arcA* strain. Notably, eGFP-tagged ribosomes were not evenly distributed, a scenario reported before [45]. We then isolated whole ribosomes as well as subunits and assessed their fluorescence intensity. In both the wild-type and ∆*arcA* strains, the 70S ribosomes and 50S subunits were fluorescent (Figure 4C). By comparing the fluorescence intensities for these two complexes between two strains, we found that the wild-type exhibited fluorescence about 1.6-fold over the *arcA* mutant. In contrast, the 30S subunits displayed fluorescence at substantially lower levels (Figure 4C), suggesting that both 50S and 30S sub-units are correctly assembled and the interaction between them (association and dissociation) appears to be independent of ArcA. All of these observations indicate that the ArcA loss impairs the ribosome biosynthesis but has no significant impact on the ribosome assembly.

### 3.5. rpsA Is under the Direct Control of ArcA

**Figure 5:**
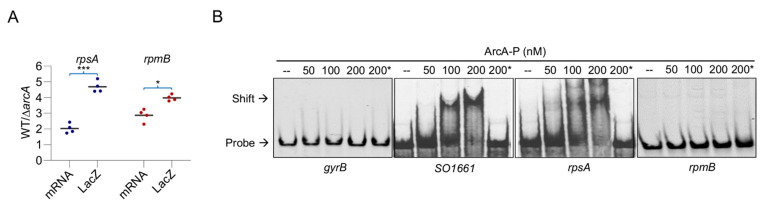
ArcA directly controls transcription of the *rpsA* gene. (**A**) Impacts of the ArcA loss on transcription and translation of *rpsA* and *rpmB*. The ratios of transcription levels revealed by qRT-PCR to translation levels revealed by *lacZ*-reporter between the wild-type and the *arcA* mutant were deduced from the data presented in Figure S5A,B. Asterisks indicate a statistically significant difference between values being compared (* *p* < 0.05; ** *p* < 0.01; *** *p* < 0.001). (**B**) Interaction of ArcA-P with promoters of interest. The EMSA assay was performed with 1 μM digoxigenin-labeled probes and various amounts of proteins as indicated. Non-specific competitor DNA (2 μM poly(dI·dC) was included in all lanes but 200*, which contains specific competitor (10 μM unlabeled probe). Promoters for GyrB and SO1661 were used as the negative and positive control respectively. Experiments were performed independently at least three times, with representative results being presented.

Although overall ribosome biosynthesis is compromised in the absence of ArcA in *S. oneidensis*, as a transcriptional regulator, ArcA impacts the physiology mainly by mediating gene expression by directly binding to promoter regions of its target genes. The DNA sequences to which *S. oneidensis* ArcA binds are highly conserved, with the consensus motif being 5-GTTAATTAAATGTTA-3 identical to that that established in *E. coli* and the ArcA regulon has been probed before by a combination of transcriptomic, in vitro DNA-protein interaction, and bioinformatics analyses [15,24,26]. According to the predicted ArcA regulon [15], the operon encoding ribosomal proteins RpsA (RSAT weight value, 11.6) is among the top 20 most confident regulon members, implying that ArcA likely influences *rpsA* transcription directly. Despite this, the difference in transcription levels of the *rpsA* gene between the wild-type and ∆*arcA* strains is approximately 2-fold revealed by the transcriptomic analysis [24], even less substantial than some of other operons for ribosomal proteins (Table S2). For verification, we compared mRNA and protein levels of the *rpsA* gene to those of the *rpmB* gene, respectively, which also encodes a ribosomal protein showing the largest changes (among genes encoding ribosomal proteins) at transcription levels caused by the ArcA loss (Table S2). Both qRT-PCR and *lacZ* reporter assays revealed that *rpsA* was transcribed and produced more than *rpmB* in both the wild-type and ∆*arcA* strains (Figure S5A,B), suggesting that RpsA may be needed in a large quantity than RpmB. However, there was more than 2-fold difference in the ratio of qRT-PCR values to *lacZ* values for both genes (Figure 5A), which is consistent with the transcriptomic and proteomic data.

Despite the similar responding patterns for *rpsA* and *rpmB*, we tested whether the *rpsA* gene is under direct control of ArcA with a DNA-binding gel shift assay. The DNA fragments, approximately 300 bp in length centered by -35 region, were used with phosphorylated ArcA (ArcA-P) because phosphorylation of ArcA is essential for its specific binding [24]. Apparently, ArcA-P significantly reduced the motility of fragments for the *rpsA* gene as well as SO_1661, a verified regulon member used as the positive control [24,26], confirming the presence of direct interaction between ArcA-P and the *rpsA* promoter sequence (Figure 5B). In contrast, no band shift was observed from either the *rpmB* gene fragments or the negative control (the GyrB gene encoding DNA gyrase sub-unit B). These results conclude that ArcA mediates expression of the *rpsA* gene directly, although its loss affects production of most, if not all, of ribosomal proteins similarly.

### 3.6. Reduced Expression of rpsA Is, at Least in Part, Accountable for Defects in Aerobic Growth but Not in the Cell Envelope Integrity Caused by the ArcA Loss

**Figure 6:**
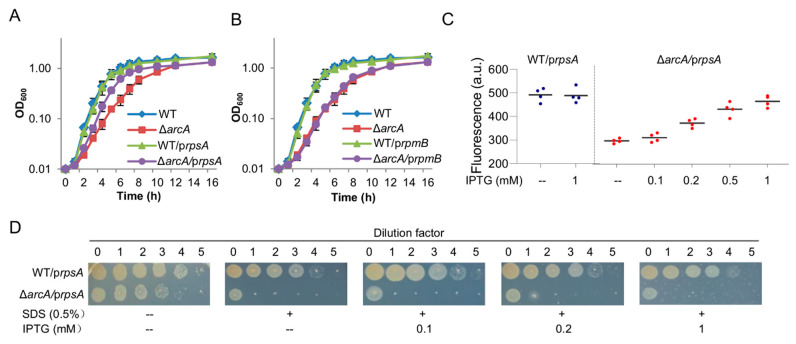
Reduced RpsA expression partially accounts for defects in aerobic growth but not the envelope. Impacts of manipulated RpsA (**A**) or RpmB (**B**) production on growth of the wild-type and ∆*arcA* strains. Growth of the wild-type and ∆*arcA* strains expressing RpsA (0.5 mM IPTG) or not in MS containing tryptone. (**C**) Fluorescence spectra of 70S ribosomal particles from the wild-type and ∆*arcA* expressing RpsA with IPTG at varying concentrations. Asterisks indicate statistically significant difference between values being compared (* *p* < 0.05; ** *p* < 0.01; *** *p* < 0.001). (**D**) SDS susceptibility revealed by droplet assay. Experiments were performed at least three times, with error bars (standard errors, *n* ≥ 3) being presented in (**A**) and (**B**), and representative data being presented in (**D**).

RpsA is traditionally called protein S1, the largest protein of bacterial ribosomes that is required for the translation initiation of mRNAs [46]. It has been established that protein S1 is not essential for ribosomal assembly although it interacts with components of head, platform, and main body of the 30S ribosomal subunit [47]. Given that transcription of the *rpsA* gene is directly controlled by ArcA, we hypothesized that the ArcA loss results in reduced production of RpsA, which leads to compromised ribosome biosynthesis. To test this, we manipulated expression of the *rpsA* gene as well as the *rpmB* gene to assess their impacts on aerobic growth when produced at varying levels. The coding sequences for the *rpsA* gene and the *rpmB* gene were placed after IPTG-inducible promoter Ptac within pHGEN-Ptac [31], and the resultant vectors were introduced into the wild-type and ∆*arcA* strains. In the wild-type, growth was not significantly altered by controlled expression of either gene with IPTG up to 1 mM (Figure 6A,B). Similar results were obtained from the ∆*arcA* strain expressing the *rpmB* gene under the same conditions (Figure 6B). However, the forced expression of the *rpsA* gene with 0.5 mM IPTG significantly improved growth of the ∆*arcA* strain (Figure 6A), suggesting that RpsA differs from RpmB in mediating ribosomal biosynthesis. It should be noted that the growth defect resulting from the ArcA loss could not be completely corrected by RpsA produced with IPTG ranging from 0.1 to 1 mM (Figure S6). Given that the Ptac promoter in the presence of 1 mM IPTG is at least 5 times stronger than the *rpsA* promoter [31,33] (Figure S5B), these data suggest that RpsA is only partially accountable for the growth phenotype.

We then attempted to determine whether the improvement in the growth of the ∆*arcA* strain by forcibly produced RpsA is a result of elevated ribosomal biosynthesis with cells producing eGFP-tagged ribosomes. In the wild-type, consistent with insignificant impacts on growth, the ribosome abundance was not significantly affected by expressing *rpsA* with 1 mM IPTG (Figure 6C). On the contrary, the ribosome abundance increased significantly in the presence of IPTG at 0.2 mM or more. Intriguingly, despite RpsA overproduction with 1 mM IPTG, the overall ribosome quantity in the *arcA* mutant appeared still lower than that in the wild-type, supporting that RpsA is not the exclusive factor for compromised ribosome biosynthesis in the ∆*arcA* strain.

We then moved on to test whether the reduced ribosome abundance caused by the *arcA* mutation also has a role in the cell envelope defect. Susceptibility of the wild-type and ∆*arcA* strains carrying vectors for *rpsA* expression to SDS was assayed. As shown in Figure 6D, the ∆*arcA* strain was hypersensitive to SDS compared to the wild-type. Despite the difference in SDS susceptibility between these two strains, the manipulated production of RpsA by up to 1 mM showed no significant impacts on their resistance to SDS, suggesting that the cell envelope defect is not associated with ribosome quantity. Therefore, the reduced ribosome abundance is not accountable for the cell envelope defect of the *arcA* mutant, further supporting the notion that two major defects caused by the ArcA loss are independent of each other [28].

## 4. Discussion

We have previously shown that the defect of an *S. oneidensis arcA* mutant in aerobic growth is dependent on tryptone [40]. As this phenomenon implies that the defect is associated with nutrients, oligopeptides in particular [42], we examined roles of peptide transporters and peptidases in *S. oneidensis*. While transport of oligopeptides and di-tripeptides is indeed to have a role in the growth phenotype, it is not the major cause [40]. Despite this, during the investigation, we noticed that expression levels of genes encoding ATP peptide transporter SapABCDF and four proton-dependent oligopeptide transporters (POTs) revealed by qRT-PCR and *lacZ* reporters differ concertedly, implying that the *arcA* mutation introduces a difference in efficacy between transcription and translation. The purpose of this study was to confirm this phenomenon and illustrate its contribution to the growth defect of the *arcA* mutant.

Given that proteins account for a large fraction of the cellular biomass and their synthesis accounts for more than two-thirds of the cell’s ATP budget during exponential growth, it is conceivable that bacterial cell growth and protein synthesis are tightly coupled [48]. Studies in systems biology have established that the RNA/protein ratio is linearly correlated with the specific bacterial growth rate allowed by the nutritional resources [12,41,49]. The data presented here and before demonstrate that the *arcA* mutation down-regulates expression of ribosomal genes and translation-related genes [24,42]. This results in low ribosome contents, verified by the discrepancy between transcription and translation of most of genes under test, by the reduced quantity of the ribosomal particles, and by that the *arcA* mutant is more susceptible to a translation-inhibiting antibiotic but not a transcription-inhibiting antibiotic. Reduction in ribosome contents in turn decreases translation of most, if not all, of genes required to maintain the wild-type growth rate, a general consequence of the ArcA loss. Nonetheless, many genes, especially those under the direct control of ArcA, are influenced predominantly at transcription levels by the ArcA loss, representing a specific response to the ArcA loss. For example, ArcA-dependent genes tested in this study and many more other ArcA regulon members, such as Dms genes, encoding for DMSO reductase and associated proteins, are dependent on ArcA for proper transcription [23,27,32].

The most well-known translation-related growth defect is carbon catabolite repression, which is mediated by the cAMP-Crp regulatory system in *E. coli* [13]. In the case, cAMP concentrations as the signal are modulated by metabolic precursors named as catabolites, the accumulation of which induces the repression. When rapidly metabolizable carbons are available, cells enhance the expression of ribosomal and anabolic genes to increase translation to support rapid cell growth [12]. *S. oneidensis* is distinct from *E. coli* in global regulators that are utilized to control respiration. This can be considered a consequence of evolution given that *Shewanella* species thrive in redox-stratified environments, where EAs are more diverse than carbon resources [19]. As *S. oneidensis* is notoriously limited in its capability to utilize carbon resources, especially six-carbon sugars [50], its global regulators probably evolve to sense and respond to EAs as we proposed before [19]. For respiration control, Fnr is negligible [16]. In contrast, cAMP-Crp has been repeatedly shown to play a predominant role, especially with respect to the respiration of non-oxygen EAs [17,18,19,20,21,22]. Nonetheless, even cAMP-Crp has rather limited impacts on the ribosome contents. This is not surprising because Crp mediates the expression of respiratory genes primarily at the transcription level, including those for a large number of cytochromes *c*, terminal reductases, and proteins required for electron transfer [15].

We have previously argued that the major role of *S. oneidensis* ArcA resides in aerobiosis on the basis that the ArcA loss does not affect growth on many non-oxygen EAs (except for DMSO) [15,23,24]. However, we were puzzled by the finding that none of proteins directly involved in oxygen respiration is subjected to regulation of ArcA in a direct manner, such as Crp-controlled cbb_3_ oxidase and cytochrome bc_1_ complex [15,19,38]. As an alternative of regulating terminal enzymes, ArcA adjusts aerobic growth mainly by mediating ribosome biosynthesis, a role that cAMP-Crp plays in *E. coli* [12]. There is a caveat for the regulation of ArcA in aerobic growth: rapid growth of *S. oneidensis* is allowed under the living conditions. There are two major types of resources that play a determining role for growth rate: nutrition and EAs. In the absence of rapidly metabolizable carbons, as in MS minimal medium, impacts of the *arcA* mutation on aerobic growth become insignificant [40]. Similarly, the *arcA* mutation does not affect growth rates when cells respire on non-oxygen EAs because growth rates under anaerobic conditions are far lower than those observed with oxygen as EA [24,42]. Seemingly, by up-regulating ribosome biosynthesis, ArcA functions to ensure cells to be able to grow rapidly when conditions are right.

Although the ArcA loss down-regulates most of ribosomal components, it is likely that only the *rpsA* gene belongs to the ArcA regulon. Among ribosomal protein components, protein S1 (RpsA) is rather unique. S1 of Gram-negative bacteria is a modular protein that contains six similar domains whereas its Gram-positive bacterial counterparts exist in many different forms [46]. Although the translation initiation of mRNAs depends on the presence of S1 [51], the protein only weakly binds to the 30S core, and as a result it is missing in the crystallographic structure of the 30S ribosomal subunit [52,53]. Our data demonstrate that RpsA, the largest ribosomal protein associated with the 30S ribosomal subunit, may have a role in the regulation of ribosomal biosynthesis. As a member of the ArcA regulon, the *rpsA* gene relies on ArcA to be transcribed to increased levels in rapidly growing cells, and it may serve as a signal for an enhanced demand of ribosome contents. This notion gains supports from the observation that the forced production of protein S1 rather than RpmB results in increased ribosome contents. Exactly how the signal cascade by which protein S1 regulates ribosome biosynthesis works need further investigation. It should be noted that the down-regulation of protein S1 production by the ArcA loss alone is not the only factor accounting for the reduced ribosome contents. We speculate that this represents an example for pleiotropic impacts of the Arc system in physiology, as it has been firmly established to be a global regulator [7,8]. Moreover, it should be noted that both initiation and elongation factors play critical roles in kinetics and fidelity of the overall translation process [54,55]. Although none of them belong to the ArcA regulon [15,24,26], further investigation into the interplays between Arc and these factors is warranted.

Although ArcA proteins have been characterized to date recognize highly conserved DNA motifs, their regulons could be profoundly different. For instance, there are only several overlaps in the *E. coli* and *S. oneidensis* ArcA regulons, which consist of at least 50 operons [7,24], suggesting that the physiological function of *S. oneidensis* ArcA is substantially different from that of the *E. coli* counterpart. In the *S. oneidensis* Arc system, the sensor protein ArcS (CaChe-PAS-PAS-HisKA) differs substantially from the *E. coli* ArcB (PAS-HisKA) in the domain structure [4,56]. However, given that *E. coli* ArcB could fully complement the ArcS and ArcS-HptA loses with respect to growth rate [25], *S. oneidensis* ArcS and *E. coli* ArcB appear to respond to similar signals to regulate the activity of ArcA. Apparently, *E. coli* ArcB is also able to activate ArcA under aerobic conditions in *S. oneidensis*. Thus, although the Arc system senses the redox signal reflecting both nutritional conditions and the presence of oxygen, its activity seems to be evolutionarily diverted to different biological processes as a means to meet specific physiological demands in each individual bacterium.

In *S. oneidensis*, another major phenotype resulting from the ArcA loss is the severe defect in the cell envelope integrity [27,28,29]. The Arc system of *E. coli* was initially recognized to confer resistance to dyes such as toluidine blue O and methylene blue, implying that the ArcA loss compromises the outer membrane, but the underlying mechanism still remains largely elusive [57]. Recently, we presented evidence to suggest that the defects in aerobic growth and the cell envelope are independent of each other [30,31]. The *arcA* mutation substantially down-regulates expression of LptFG genes, which encode two essential components of lipopolysaccharide transport (Lpt) system, resulting in a defect in the cell envelope [29]. This defect can be alleviated only when σ^E^, a master regulator establishing and maintaining the integrity of in bacteria, is present to activate cell envelope stress response [28,29]. In this study, we found that the forced production of RpsA has no influence on the cell envelope defect of the *arcA* mutant, eliminating a possibility that the reduction in the ribosome contents also significantly affects the cell envelope biogenesis.

## Figures and Tables

**Table 1:** Strains and plasmids used in this study.

Strain or Plasmid	Description	Reference or Source
*E. coli* strains		
DH5α	Host for cloning	Lab stock
WM3064	Δ*dapA*, donor strain for conjugation	W. Metcalf, UIUC
*S. oneidensis* strains		
MR-1	Wild type	ATCC 700550
HG0624	∆*crp* derived from MR-1	[15]
HG2356	∆*fnr* derived from MR-1	[15]
HG3988	∆*arcA* derived from MR-1	[24]
HG3927W	eGFP knock-in at *rplI* derived from MR-1	This study
HG3927A	eGFP knock-in at *rplI* derived from ∆*arcA*	This study
Plasmid		
pHGM01	Ap^r^ Gm^r^ Cm^r^ suicide vector	[31]
pHGEI01	Km^r^, integrative *lacZ* reporter vector	[32]
pHGEN–Ptac	Km^r^, IPTG-inducible expression vector	[33]
pBBR–Cre	Sp^r^, helper plasmid for antibiotic cassette removal	[18]
pHG101–ArcA	Complementation vector carrying *arcA*	[29]
pHGEI01–PaprE	PaprE–lacZ fusion within pHGEI01	[29]
pHGEI01–Psap	Psap–lacZ fusion within pHGEI01	This study
pHGEI01–PdtpA	PdtpA–lacZ fusion within pHGEI01	This study
pHGEI01–PdtpB	PdtpB–lacZ fusion within pHGEI01	This study
pHGEI01–PSO1505	PSO1505–lacZ fusion within pHGEI01	This study
pHGEI01–PSO3195	PSO3195–lacZ fusion within pHGEI01	This study
pHGEI01–PrplK	PrplK–lacZ fusion within pHGEI01	This study
pHGEI01–PsspA	PsspA–lacZ fusion within pHGEI01	This study
pHGEI01–PSO0783	PSO0783–lacZ fusion within pHGEI01	This study
pHGEI01–PbtuD	PbtuD–lacZ fusion within pHGEI01	This study
pHGEI01–PnuoA	PnuoA–lacZ fusion within pHGEI01	This study
pHGEI01–PSO2061	PSO2016–lacZ fusion within pHGEI01	This study
pHGEI01–PSO3282	PSO3282–lacZ fusion within pHGEI01	This study
pHGEI01–PSO4542	PSO4542–lacZ fusion within pHGEI01	This study
pHGEI01–PpspF	PpspF–lacZ fusion within pHGEI01	This study
pHGEI01–PhoxK	PhoxK–lacZ fusion within pHGEI01	This study
pHGEI01–PcsgB	PcsgB–lacZ fusion within pHGEI01	This study
pHGEI01–PrpsA	PrpsA–lacZ fusion within pHGEI01	This study
pHGEI01–PrpmB	PrpmB–lacZ fusion within pHGEI01	This study
pHGEN-Ptac-rpsA	Ptac-rpsA within pHGE-Ptac	This study
pHGEN-Ptac-rpmB	Ptac-rpmB within pHGE-Ptac	This study

## Data Availability

Most of the data presented in this study are included in this published article and in the Supplementary Materials. Additional data, including primer sequence information and raw data for growth curves, generated or analyzed during the current study are available from the corresponding author on reasonable request.

## Supplementary Materials

The following are available online at https://www.mdpi.com/article/10.3390/life11090926/s1, Figure S1: Different expression of oligopeptide transporter genes revealed by qRT-PCR and lacZ-reporters. Figure S2: Defects of the ArcA mutant in aerobic growth. Figure S3: Transcription and translation of 12 genes in the wild-type, ∆*fnr*, and ∆*crp* strains. Figure S4: L9 fusions do not affect growth. Figure S5: Impacts of the manipulated production of RpsA or RpmB loss on transcription and translation. Figure S6: Effects of manipulated RpsA production on growth of the ArcA mutant. Table S1: Twelve genes whose transcription and translation were studied. Table S2: Differences in expression of ribosomal proteins revealed by microarrays and proteomics analysis.
