# Supplementary material for: Shewanella oneidensis arcA Mutation Impairs Aerobic Growth Mainly by Compromising Translation
Source: Life (Basel). 2021 Sep 6;11(9):926. doi: 10.3390/life11090926 (PMC8470723; doi:10.3390/life11090926)
Supplement: Supplementary file 1 [file life-11-00926-s001.zip › life-1365445-supplementary.pdf]

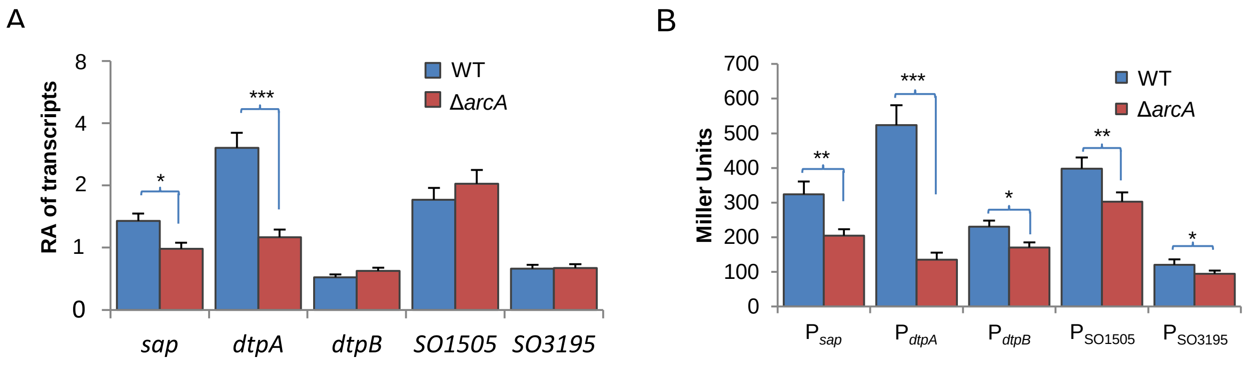

FIGURE S1. Different expression of oligopeptide transporter genes revealed by qRT-PCR and *lacZ*-reporters. (A) Expression levels revealed by qRT-PCR assay. The wild-type and Δ*arcA* strains grown to the mid-log phase in MS containing 0.5% tryptone were used for the assay. The averaged values for each gene under test were normalized to that of the *recA* gene, giving to relative abundance (RA) of transcripts. Asterisks indicate statistically significant difference (*, $P < 0.05$; **, $P < 0.01$; ***, $P < 0.001$). (B) Promoter activity assay. Indicated promoters were used to drive expression of the full-length *E. coli lacZ* gene within an integrative system. β-galactosidase activities in the mid-log phase cells were determined and presented as Miller Units. Experiments were performed independently at least 5 times, and data were presented as the average and error bars representing standard errors.

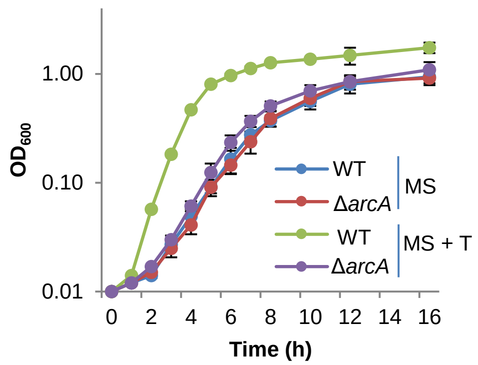

FIGURE S2. Defects of the *arcA* mutant in aerobic growth. Growth of the wild-type and Δ*arcA* strains in MS or MS containing 0.5% tryptone (T). Experiments were performed independently at least 5 times, and data were presented as the average and error bars representing standard deviation.

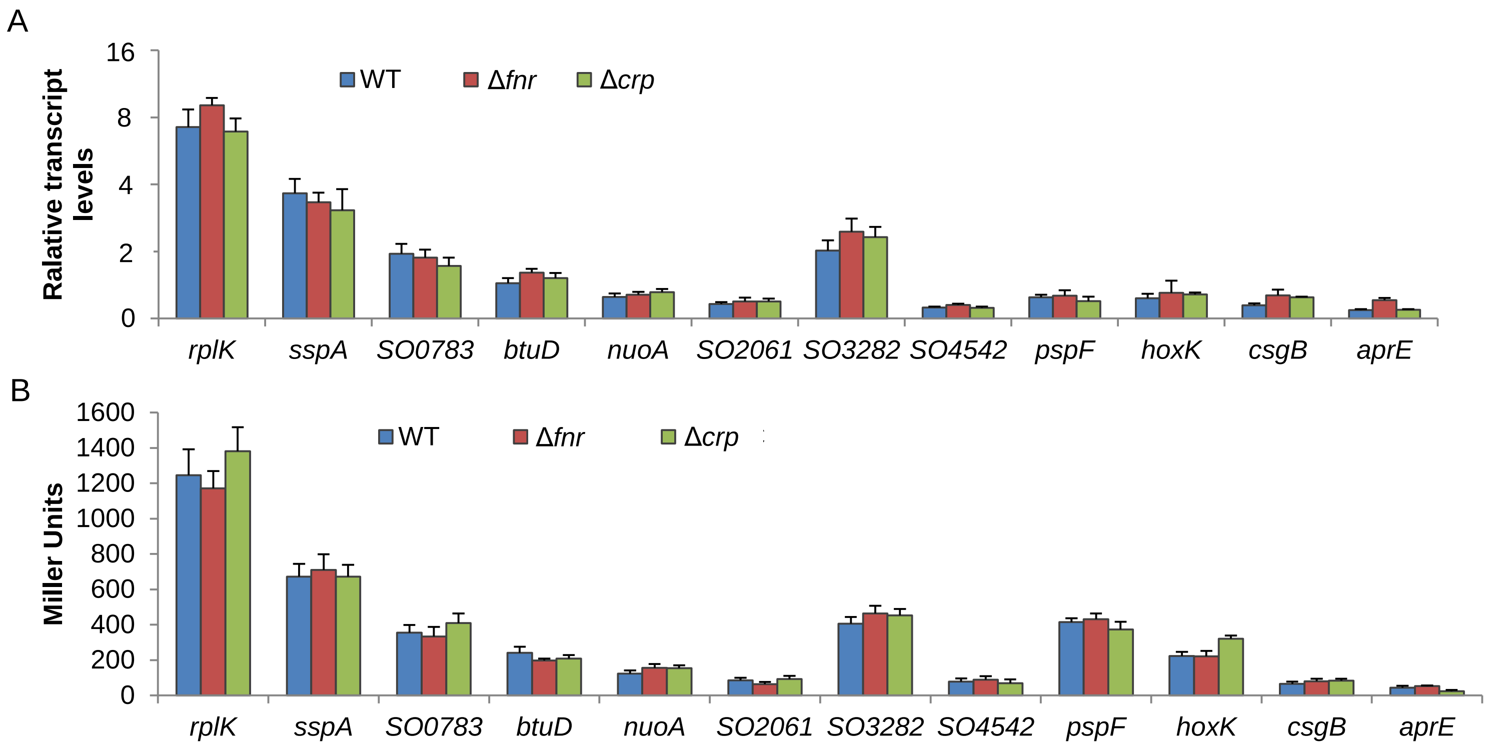

FIGURE S3. Transcription and translation of 12 genes in the wild-type, Δ*fnr*, and Δ*crp* strains. (A) Transcription levels revealed by qRT-PCR assay. Promoters of indicated genes were used to drive expression of the full-length *E. coli lacZ* gene integrated in the wild-type and Δ*arcA* strains, which were grown to the mid-log phase in MS containing 0.5% tryptone. The averaged values for *lacZ* transcripts were normalized to that of the *recA* gene, giving to relative abundance (RA) of transcripts. (B) Promoter activity assay. β-galactosidase activities in cells used in (A) were determined and presented as Miller units. Experiments were performed independently at least 3 times, and data were presented as the average and error bars representing standard errors.

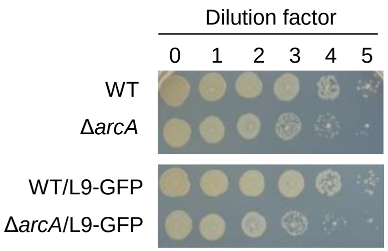

FIGURE S4. L9 fusions do not affect growth. Growth determined by droplet assays on MS + tryptone plates. Experiments were performed independently at least 3 times, with representative results being presented.

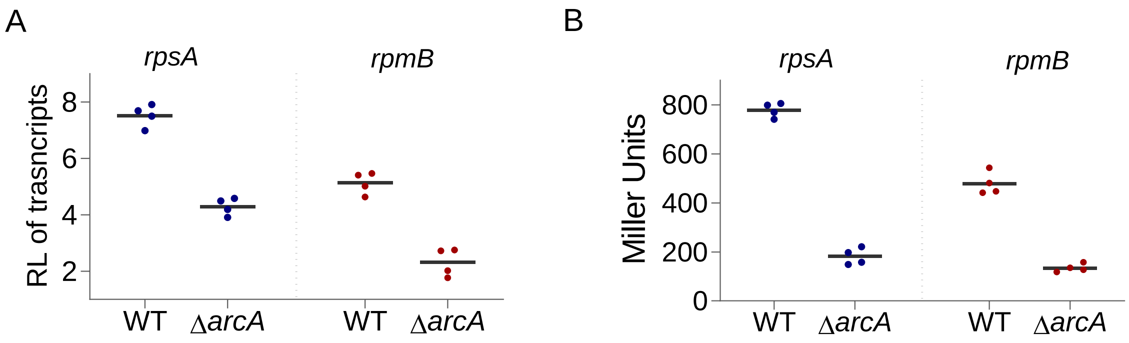

FIGURE S5. Impacts of the manipulated production of RpsA or RpmB loss on transcription and translation. (A) Transcription levels of 12 genes revealed by qRT-PCR assay. (B) Promoter activity assay. β-galactosidase activities in cells used in (A) were determined and presented as Miller units. Experiments were carried out the same as in Fig. 1.

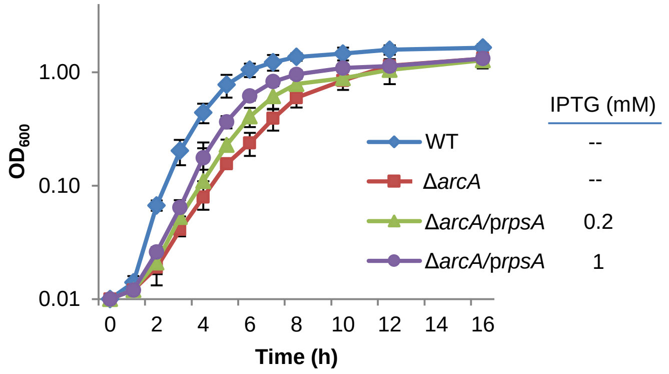

FIGURE S6. Effects of manipulated RpsA production on growth of the *arcA* mutant. Growth was determined by liquid MS + tryptone. Experiments were performed independently at least 3 times, and data were presented as the average and error bars representing standard deviation.

TABLE S1. Twelve genes whose transcription and translation were studied

locus	Gene	Annotated function	ArcA-binding motif[a]
SO_0220	*rplk*	50S ribosomal protein L11	
SO_1806	*pspF*	psp operon transcriptional activator	GTTAATAAAATGTTT
SO_0611	*sspA*	transcpritional activator	
SO_0783		superfamily I DNA and RNA helicase	
SO_0815	*btuB*	ABC-type cobalamin uptake system ATPase component	
SO_0866	*csgB*	minor curlin subunit	GTAATTTAAATGTTA
SO_1021	*nuoA*	NADH-quinone oxidoreductase subunit A	
SO_2061		predicted lipoprotein	
SO_2099	*hoxK*	quinone-reactive NiFe hydrogenase, small subunit	GTTAATTAAATGTCA
SO_3106	*aprE*	extracellular serine alkaline protease	GTAAATTAATTGTTA
SO_3282		energy taxis modulating methyl accepting sensory transducer	
SO_4542		transcriptional regulator LysR family	

[a] genes with an ArcA-binding motif are members of the ArcA regulon.

TABLE S2. Differences in expression of ribosomal proteins revealed by microarrays and proteomics analysis[a]

Ribosomal components	Locus (gene)	Transcriptomics Log2(Δ*arcA*/WT)	Proteomics Log2(Δ*arcA*/WT)
LSU L1P	SO_0221 (RplA)	-1.4	-1.1
LSU L2P	SO_0234 (RplB)	-0.1	-1.3
LSU L3P	SO_0231 (RplC)	-1.0	-1.5
LSU L4P	SO_0232 (RplD)	-0.1	-1.0
LSU L5P	SO_0243 (RplE)	-1.4	-1.3
LSU L6P	SO_0246 (RplF)	-0.6	-1.5
LSU L9P	SO_3927 (RplI)	-1.1	-1.8
LSU L10P	SO_0222 (RplJ)	-1.2	-1.9
LSU L11P	SO_0220 (RplK)	-1.4	-1.5
LSU L7/12P	SO_0223 (RplL)	-1.3	-2.7
LSU L13P	SO_3940 (RplM)	-0.9	-1.2
LSU L14P	SO_0241 (RplN)	-1.2	-1.6
LSU L15P	SO_0250 (RplO)	-0.5	-1.4
LSU L16P	SO_0238 (RplP)	-0.7	-1.4
LSU L17P	SO_0257 (RplQ)	-0.9	-1.4
LSU L18P	SO_0247 (RplR)	-0.4	-1.8
LSU L19P	SO_1360 (RplS)	-1.0	-1.3
LSU L20P	SO_2302 (RplT)	-0.9	-0.5
LSU L21P	SO_3652 (RplU)	-0.6	-1.2
LSU L22P	SO_0236 (RplV)	-0.4	-1.9
LSU L23P	SO_0233	-0.9	-1.2
LSU L24P	SO_0242 (RplW)	-1.3	-1.5
LSU L25P	SO_2112	-0.8	-1.3
LSU L27P	SO_3651 (RpmA)	-0.9	-1.8
LSU L28P	SO_4247 (RpmB)	-1.6	-2.1
LSU L29P	SO_0239 (RpmC)	0.0	-1.5
LSU L30P	SO_0249 (RpmD)	-0.9	-1.9
LSU L31P	SO_4120 (RpmE)	-0.4	-2.0
LSU L32P	SO_2780 (RpmF)	-0.9	-1.6
LSU L33P	SO_4246 (RpmG)	-1.6	-2.2
LSU L34P	SO_0007 (RpmH)	-0.8	No Data
LSU L35P	SO_2301 (RpmI)	0.0	No Data
LSU L36P	SO_0252 (RpmJ)	-1.2	No Data
SSU S1P	SO_2402 (RpsA)	-1.0	-2.3
SSU S2P	SO_1629 (RpsB)	-1.2	-2.0
SSU S3P	SO_0237 (RpsC)	-0.4	-0.8
SSU S4P	SO_0255 (RpsD)	-0.6	-2.0
SSU S5P	SO_0248 (RpsE)	-1.0	-2.3
SSU S6P	SO_3930 (RpsF)	-0.8	-1.9
SSU S7P	SO_0227 (RpsG)	-1.2	-0.7

SSU S8P	SO_0245 (RpsH)	-0.9	-2.0
SSU S9P	SO_3939 (RpsI)	-1.4	-0.7
SSU S10P	SO_0230 (RpsJ)	-1.3	-0.9
SSU S11P	SO_0254 (RpsK)	-0.9	-2.1
SSU S12P	SO_0226 (RpsL)	-1.5	-1.5
SSU S13P	SO_0253 (RpsM)	-0.7	-0.2
SSU S14P	SO_0244 (RpsN)	-0.9	-1.1
SSU S15P	SO_1207 (RpsO)	-0.8	-1.4
SSU S16P	SO_1357 (RpsP)	-1.2	-2.9
SSU S17P	SO_0240 (RpsQ)	-0.6	-0.8
SSU S18P	SO_3928 (RpsR)	-1.4	-1.4
SSU S19P	SO_0235 (RpsS)	-0.5	-0.8
SSU S20P	SO_3537 (RpsT)	-0.2	-1.0
SSU S21P	SO_1288 (RpsU)	-0.1	-2.3

[a] Transcriptomics and proteomics data are from references 24 and 42, respectively.
